# A phase I/II clinical trial on the efficacy and safety of NKT cells combined with gefitinib for advanced EGFR-mutated non-small-cell lung cancer

**DOI:** 10.1186/s12885-021-08590-1

**Published:** 2021-07-31

**Authors:** Wanjun Yu, Fei Ye, Xiao Yuan, Yali Ma, Chaoming Mao, Xiaoqin Li, Jian Li, Chunhua Dai, Fenhong Qian, Junrong Li, Xiujuan Fan, Yuepeng Zhou, Deqiang Wang, Zhenhong Guo, Huazhang An, Minghui Zhang, Deyu Chen, Sheng Xia

**Affiliations:** 1 Department of Immunology, School of Medicine, Jiangsu University, Zhenjiang, China; 2 Department of Oncology, The Affiliated Hospital of Jiangsu University, Zhenjiang, China; 3 Department of Nuclear Medicine, The Affiliated Hospital of Jiangsu University, Zhenjiang, China; 4 Department of Respiratory, The Affiliated Hospital of Jiangsu University, Zhenjiang, China; 5 Department of Epidemiology and Biostatistics, School of Medicine, Jiangsu University, Zhenjiang, China; 6 National Key Laboratory of Medical Immunology & Institute of Immunology, Second Military Medical University, Shanghai, China; 7 Shandong Provincial Qianfoshan Hospital, Shandong First Medical University, Jinan, Shandong China; 8 School of Medicine, Tsinghua University, Beijing, China

**Keywords:** Non-small-cell lung cancer, Natural killer T cell, Immunotherapy, Gefitinib, Randomized controlled trial

## Abstract

**Background:**

Epidermal growth factor receptor tyrosine kinase inhibitors (EGFR-TKIs), such as gefitinib, have achieved good efficacy in EGFR mutation-positive non-small-cell lung cancer (NSCLC) patients, but eventual drug resistance is inevitable. Thus, new TKI-based combination therapies should be urgently explored to extend the overall survival time of these patients. CD8 + CD56+ natural killer T (NKT) cells are a natural and unique subset of lymphocytes in humans that present characteristics of T and NK cells and exert cytotoxicity on tumour cells in a granzyme B-dependent manner. The aim of this trial was to explore the efficacy and safety of CD8 + CD56+ NKT cell immunotherapy combined with gefitinib in patients with advanced EGFR-mutated NSCLC.

**Methods:**

The study was designed as a prospective, randomized, controlled, open-label, phase I/II trial that includes 30 patients with EGFR mutation-positive stage III/IV NSCLC. All patients will be randomized in blocks at a 1:1 ratio and treated with gefitinib 250 mg/day monotherapy or combination therapy with allogeneic CD8 + CD56+ NKT cell infusions twice per month for 12 cycles or until disease progression occurs. The effectiveness of this treatment will be evaluated based on by progression-free survival (PFS), the time to progression (TTP), overall response rate (ORR), disease control rate (DCR) and overall survival (OS). The safety of the trail is being assessed based on adverse events (AEs). Recruitment and data collection, which started in December 2017, are ongoing.

**Discussion:**

Although immunotherapy, including programmed death-1/programmed death-1 ligand (PD-1/PD-L1) immunotherapy, has been used for NSCLC treatment with or without EGFR-TKIs, its clear efficacy still has not been shown. Assessing the safety and therapeutic potential of allogeneic CD8 + CD56+ NKT killer cells in combination with EGFR-TKIs in NSCLC will be of great interest.

**Trial registration:**

This trial (Phase I/II Trails of NKT Cell in Combination With Gefitinib For Non Small Cell Lung Cancer) was registered on 21 November 2017 with www.chictr.org.cn, ChiCTR-IIR-17013471.

**Supplementary Information:**

The online version contains supplementary material available at 10.1186/s12885-021-08590-1.

## Background

### EGFR-TKI and NSCLC

According to a status report on the worldwide cancer burden in 2018, lung cancer remains the leading cause of cancer incidence and mortality [1]. Among the approximately 2 million new cases each year, 80–85% of new cases are non-small-cell lung cancer (NSCLC), such as adenocarcinoma, squamous cell carcinoma, and large cell carcinoma, and 15–20% are small-cell lung cancer. Platinum chemotherapy has been the main systemic therapy for patients with advanced NSCLC over the past few decades [2]. In 2004, published data showed that somatic mutations in the epidermal growth factor receptor (EGFR) gene are detected in 30–40% of NSCLCs in Asian patients, and deletions in exon 19 and L858R in exon 21 account for 90% of these activating EGFR mutations [3]. Several epidermal growth factor receptor tyrosine kinase inhibitors (EGFR-TKIs), such as gefitinib, have shown a higher response rate and longer progression-free survival (PFS) than platinum chemotherapy in patients with EGFR mutations and have become a first-line treatment for advanced NSCLC [4, 5]. Thus, these key studies clarified the relationship between EGFR mutations and the efficacy of EGFR-TKI treatment, providing opportunities for the personalised treatment of advanced NSCLC. Though the majority of patients with mutation-positive NSCLC respond to treatment with EGFR-TKIs, the long-term use of EGFR-TKIs for approximately 9–12 months eventually leads to drug resistance, which limits their clinical benefits [6–8]. In addition, co-mutation is common in NSCLC patients with EGFR mutations, and the efficacy of first-line TKI monotherapy in NSCLC patients with EGFR co-mutations is significantly worse than that in patients without co-mutations [9]. Therefore, for EGFR mutation-positive patients, TKI-based combination therapy, especially with immunotherapy, needs to be further explored [10].

### Immunotherapy and NSCLC

Immunotherapy is a novel, promising treatment for cancers that uses different effective elements of the immune system, such as cytokines, antibodies and effector immune cells. Currently, immune checkpoint inhibitors, anti-programmed death-1 (anti-PD-1) and anti-programmed death-1 ligand (anti-PD-L1), have shown promising effects for treating tumours [11]. Several combination trials of EGFR-TKIs and immune checkpoint inhibitors in NSCLC are also recruiting participants. Nevertheless, some evidence has shown that anti-PD-1 therapy has no additional effects with EGFR-TKI treatment because of the lower expression levels of PD-1 among EGFR-mutant NSCLC patients [12, 13]. Therefore, more evidence is needed on the actual synergistic effects of anti-PD-1 and anti-PD-L1 with EGFR-TKIs [14, 15].

## Rationale

In the immune system, there are different types of killer cells that play roles in immune surveillance. Therefore, methods to isolate these cells from the blood, culture and expand them with or without gene editing in the laboratory, and finally infuse these killer cells into the body of patients to exert anti-tumour effects have also been designed for cancer immunotherapy. Currently, the types of killer cells used in cellular immunotherapy are CD8+ T cells including tumour-infiltrating lymphocytes (TILs) and chimeric antigen receptor T (CAR-T) cells, NK cells, and natural killer T (NKT) cells. NKT cells are a subset of CD1d-restricted T cells at the interface between the innate and adaptive immune systems and have characteristics of both conventional T cells and NK cells [16]. Classical NKT cells with invariant TCR α chains were first identified in mice in 1990 and subdivided into different subsets that have a variety of roles in cancer and other diseases [17, 18]. Nevertheless, in some studies, NKT cells have also been identified with flow cytometry analysis with the cell phenotype of CD3 + CD56+ or CD8 + CD56+ in humans and CD3 + NK1.1+ or CD8 + NK1.1+ in mice. Though the definition of these CD3+ subpopulations as NKT cells is still debatable, and they are even referred to as “NKT-like” cells, these cells show activity in innate immunity [19–23]. The work from Sergey S. Seregin showed that CD8 + NK1.1+ cells, not CD8 + NK1.1- cells, have the ability to provide a rapid innate immune response through Ag-independent IFN-γ production and granzyme B degranulation in pathogen infection [22, 23]. Our previous data also demonstrated that CD8 + NK1.1+ cells exerted NK- and CTL-like antitumour effects through the elimination of both tumour cells and myeloid-derived suppressor cells (MDSCs) in a granzyme B-dependent manner [24]. Moreover, we further proved that CD8 + CD56+ cells in humans have similar cytotoxicity to CD8 + NK1.1+ cells in mice and can be effectively expanded in vitro for tumour immunotherapy. However, it is unclear whether these CD8 + CD56+ NKT cells have synergistic effects with EGFR-TKIs, extend PFS and delay resistance to EGFR-TKIs in NSCLCs. Thus, a trial assessing the efficacy of CD8 + CD56+ NKT cells with gefitinib for patients with EGFR mutations is needed.

## Methods

### Objectives

The aim of this trial is to assess whether the proposed allogeneic NKT cell adoptive transfer, when given in combination with gefitinib, warrants further consideration based on its safety and efficacy for the treatment of advanced EGFR-mutant NSCLCs. Effectiveness is being evaluated by PFS, the time to progression (TTP), overall response rate(ORR), disease control rate (DCR) and overall survival (OS). Safety assessment is based on adverse events (AEs), vital signs, clinical laboratory tests, such as haematologic, blood biochemical and urine analysis, and electrocardiograms.

### Trail design

The study protocol (Issue date: 9 September 2017, protocol amendment number: v1.2) was written in accordance with the Standard Protocol Items: Recommendations for Interventional Trials (SPIRIT) 2013 Statement [25]. A SPIRIT checklist is provided in the supplementary material (Additional file 1: Table S1). This early exploratory phase I/II clinical trial was initiated by the investigators and supported by the key research and development special funding of the Jiangsu Science and Technology Bureau for social development. The affiliated Hospital of Jiangsu University is responsible for clinical research.

**Fig. 1:**
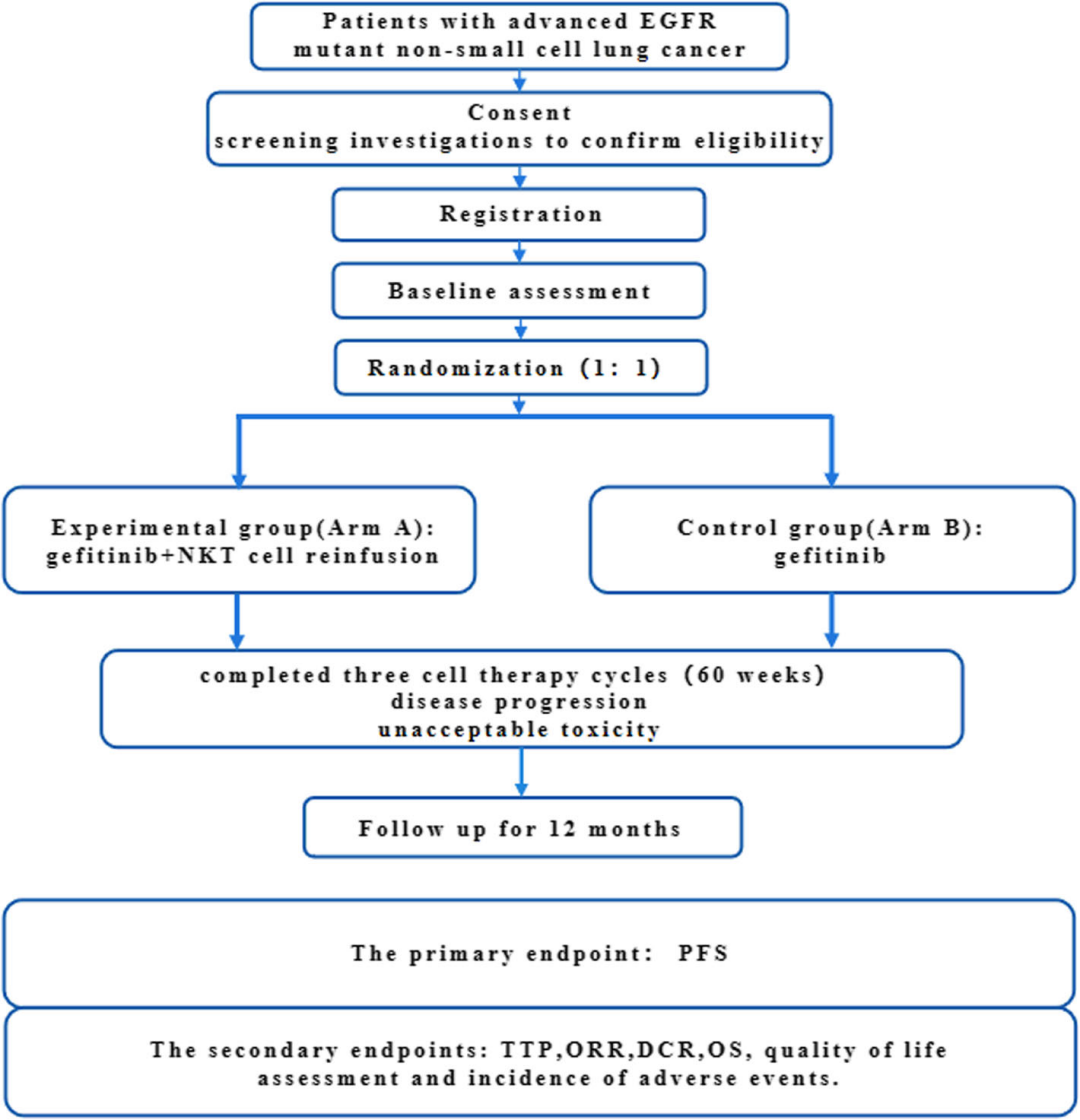
Flow diagram of this parallel randomised trial. EGFR: epidermal growth factor receptor; PFS: progression-free survival; TTP: time to progression; ORR: overall response rate; DCR: disease control rate; OS: overall survival

The study was designed as a prospective, randomized, open-label, controlled phase I/II parallel-group trial to explore the efficacy and safety of NKT cell infusion immunotherapy combined with gefitinib versus gefitinib monotherapy in patients with advanced EGFR-mutated NSCLC (Fig. 1). This study will recruit 30 participants with advanced NSCLC who meet the inclusion/exclusion criteria and individually randomize them at a 1:1 ratio based on the trial schedule (Fig. 1). The two arms of the trial are as follows (Table 1):

- Experimental group (Arm A): 250 mg gefitinib orally once daily. After being effectively treated with gefitinib for 8 weeks, allogeneic NKT cell transfer treatment will be added every 4 weeks, with a total cell number of 1 × 10^10^ ± 15% divided into two infusions with an interval of 3 days. This process will be repeated for a total of 16 weeks, which is defined as one-cell therapy cycle, and then NKT cell infusion treatment will be suspended for 4 weeks. After that, this cycle will be continually repeated three times (total of 60 weeks) or stopped when disease progression/unacceptable toxicity/participant choice occurs (Table 1).
- Control group (Arm B): 250 mg gefitinib orally once daily until disease progression/unacceptable toxicity/participant choice occurs.

**Table 1:** Treatment schedule for the frequency of gefitinib and NKT cell in both arms of the trail

Week-4-0	Week 0–8			One cell therapy cycle				
				Week 9–12	Week 13–16	Week 17–20	Week 21–24	Week 25–28
Screening	Randomization	Experimental group (Arm A)	Donor selection, PBMCs^a^ acquisition and NKT cell preparation	NKT infusion	NKT infusion	NKT infusion	NKT infusion	Suspend
			Gefitinib orally once daily					
		Control group (Arm B)	Gefitinib orally once daily					

This schedule use one cell therapy cycle as an example, and the follow-up treatment is carried out according to the same cell therapy cycle until the trial is terminated after the disease has progressed or complete three cell therapy cycles (total 60 weeks). ^a^*PBMCs* peripheral blood mononuclear cells

### Study population

The main participant inclusion and exclusion criteria are shown in Table 2. This trial aims to include only patients with advanced NSCLC (III/IV) harbouring mutated EGFR (exon 19 deletion or exon 21 Leu858 Arg point mutation) who can be effectively treated with gefitinib.

**Table 2:** Eligibility criteria

Key inclusion criteria	Key exclusion criteria
• Histological diagnosis of advanced non-small cell lung cancer (III/IV) with measurable lesions; • No other chemotherapy and radiation therapy to be planned recently. I • Aged 18 to 75 years, male or female; • Evidence of activating mutations of EGFR(exon 19 deletion or exon 21 Leu858Arg point mutation)and can be treated with gefitinib; • Patients must have a Karnofsky performance status greater than or equal to 80%; • There is no disease progression after being treated with gefitinib for 8 weeks; • Adequate bone marrow reserve and adequate live and renal functions: hemoglobin ≥9 g/dL, absolute neutrophil (segmented and bands) count (ANC) ≥ 1.5 × 10^9^/L, lymphocytes count ≥ lower limit of normal reference value, platelet count ≥8 × l0^10^/L, serum creatinine ≤1.5 × high limit of normal reference value, Serum bilirubin ≤2 × upper limit of normal reference value, both of AST/ALT ≤2 × upper limit of normal reference value; • Women of childbearing potential must have a negative pregnancy test (within 7 days) prior to receiving treatment of study agent; • Life expectancy greater than 12 months; • Patients have ability to understand and subscribe of informed consent.	• Organ dysfunction defined as follows: significant cardiovascular disease (i.e. New York Heart Association [NYHA] class 3 congestive heart failure, myocardial infarction within the past 6 months, unstable angina, coronary angioplasty within the past 6 months, uncontrolled atrial or ventricular cardiac arrhythmias); Liver function grading Child-Pugh C; renal function failure or uremia; respiratory failure; disturbance of consciousness; • Patients with genetic diseases; • Known central nervous system tumors including metastatic brain disease, unless treated and stable; • Suffering from lymphoma, leukemia and myelodysplastic syndrome (MDS); • Serious infections requiring antibiotics treatment, bleeding disorders; • History of bone marrow or stem cell transplantation, or allograft transplantation; • Patients with immunodeficiency disease or autoimmune disease, except vitiligo; • Patients with allergy history, especially allergy to heterologous proteins; • Uncontrolled infectious diseases and other serious diseases, such as patients with HIV positive, active HBV and HCV hepatitis; • Patients with chronic disease which is undergoing immune reagents or hormone therapy (Topical or inhalational corticosteroids are permitted); • Patients with concurrent chemotherapy or in five half-life periods of the used chemotherapy drugs; • Pregnant or breast-feeding women; • Mental impairment or addictive disorders that may interfere the ability to sign informed consent; • Lack of availability for immunological and clinical follow-up assessment.

This is also an exploratory study to test the safety and efficacy of the intravenous infusion of CD8 + CD56+ NKT cells, which replicates a planned larger study in at a smaller scale. Some authors have put forward suggestions on the sample size of pilot trials, specifically, 10 [26], 12 [27], or 15 [28] per group and a total of at least 20 participants [29]. According to a sample size “rule of thumb” of 30, the number of patients was designated as 15 patients in the experimental group and 15 patients in the control group, with a total sample size of 30 patients in this study. Although the power of this study is inadequate for evaluating clinical efficacy, the estimation of the efficacy of NKT cell immunotherapy combined with gefitinib and gefitinib alone will enable follow-up studies to calculate the efficacy more accurately.

### Randomization

After the subjects are registered (and before any trial treatment), the subjects will be randomly assigned to the experimental group (Arm A) or the control group (Arm B) at a proportion of 1:1. The method of block randomization will be used to randomize the subjects into the groups. The random number table will be generated by statistics experts.

### Preparation and administration of NKT cells

In this study, healthy immediate family members of the patient will be selected as donors for peripheral blood of nuclear cell (PBMC) collection and NKT cell preparation. PBMCs of the donor will be collected by an automatic blood cell separator and then frozen in liquid nitrogen. In the treatment phase of the study, PBMCs will be thawed, cultured and expanded with NKT complete medium with a protocol specially developed by the Institute of Cell Therapy, Tsinghua University, China. Prior to cell infusion, the quality of expanded NKT cells will be tested based on the cell number, purity, viability and sterility. The criteria of the tested cells included viability ≥90%, CD8+ ≥60%, and a minimum of 20% CD8 + CD56+ NKT cells. Along with gefitinib treatment in both Arms A and B, all patients in Arm A will intravenously receive 1 × 10^10^ expanded cells over 1 month with an interval of 3 days according to the schedule in Table 1. At the end of the cell transfusion, the subject will lie still and will be observed for 1 h, and their normal activities can be resumed if there is no discomfort or other adverse reactions.

## Assessments

A schedule of procedures and assessments is shown in Additional file 2: Table S2.

Several assessments will be used for the evaluation of therapeutic effects, including tumour lesion measurements, patient quality of life scores, and the detection of serum tumour markers, such as CEA. For tumour lesion detection, according to the Response Evaluation Criteria in Solid Tumors version 1.1 (RECIST Version 1.1), computed tomography (CT) will be used to measure the size of solid tumours during screening, treatment and follow-up in the study. All baseline assessments of the tumour focus size should be completed as close to the start of treatment as possible. Additionally, the KPS score and EORTC QLQ-C30-LC13 score will be evaluated to assess the quality of life of patients with lung cancer in all stages.

AEs, vital signs and safety laboratory examinations will be used for safety evaluation. The severity of AEs will be graded using a modified version of The Common Terminology Criteria for AEs (CTCAE v5.0), reported and addressed at any time during the course of the study. Nevertheless, vital signs, including blood pressure, body temperature, respiration and heart rate, will be monitored during all visits, and routine safety laboratory examinations are detailed in Table S2.

## Outcomes

### Primary effectiveness endpoint

The efficacy will be evaluated based on PFS, which is defined as the time from randomization to the first recording of disease progression (as defined by RECIST v1.1) or the death of the patient. Patients who are still alive and have no progression as of the date of analysis will be recorded as the date of their last imaging evaluation.

### Secondary effectiveness endpoints

TPP: the length of time from the date of randomization to tumour progression based on RECIST v1.1 criteria.

ORR: the proportion of participants with complete remission and partial remission, as judged by RECIST v1.1.

DCR: the proportion of participants with an objective response and stable disease, as defined by RECIST v1.1.

OS: defined as the time from the beginning of randomization to death from any cause. For patients who are still alive on the date of analysis, the date of their last contact will be recorded as the endpoint.

### Security endpoints

Safety and toxicity assessment will be calculated based on AEs, vital signs, clinical laboratory tests, and electrocardiograms. Each AE will be monitored during the study, and its nature, intensity and relationship with treatment will be evaluated.

## Statistical analysis

A full analysis set will be used to analyse the primary and secondary efficacy indicators. The full analysis set refers to the ideal subject set, which is as close as possible to intention-to-treat principles (the main analysis should include all subjects). For missing value estimation of the main variables, the last observation carried forward (LOCF) method will be used to carry forward the missing data of the study, and the number of subjects whose efficacy is evaluated at the endpoint will be consistent with that at the beginning of the study. The safety set is the main group of safety evaluations in this study. All groups with at least one study drug (cell preparation) and at least one safety assessment record will constitute the safety set of this study.

All statistical tests will be conducted using unilateral tests, and *P* < 0.05 will be considered to be statistically significant (except when specifically stated). Measurement data will be statistically described by the mean, median, standard deviation, maximum, minimum, and 25 and 75% quantiles; counting data or grade data will be expressed as the frequency. FPS, TTP and OS will be estimated by the Kaplan-Meier method and compared between groups with an unstratified log-rank test. Greenwood’s formula will be used to calculate 95% CIs. Fisher’s exact probability method will be used to compare the incidence of ORR, DCR and AEs between the two groups. The Wilcoxon rank-sum test will be used to compare the significant differences between the two groups for adverse reactions of different degrees. Changes in laboratory test results and quality of life assessments during the study will be summarized in tables and graphs. In addition, the relationship between abnormal changes and cell preparation will be further analysed.

## Discussion

The ideal combination of immunotherapy with EGFR-TKIs should target different mechanisms of tumour-induced immunosuppression in the microenvironment [30]. Different types of killer cells combined with surgery and chemoradiotherapy have been explored for immunotherapy in NSCLC, including TILs and dendritic cell-cytokine induced killer (DC-CIK) cells [31–33]. Gefitinib prevents the proliferation of mutant EGFR-dependent cells, enhances the presentation of tumour antigens, and promotes the apoptosis of tumour cells without affecting biological functions of immune effector cells. However, as described in previous publications, the efficacy of autologous killer cell infusion in the clinic still needs to be improved [34]. In this study, we used allogeneic CD8 + CD56+ killer cells combined with gefitinib to treat NSCLC with EGFR mutations. Compared with previous studies, there are several different properties of this clinical design. First, the cells we used in this protocol are different from previously reported DC-CIK cells, which are rich in CD8 + CD56+ NKT cells. The biological properties of CD8 + CD56+ cells in humans and their equivalent subset, CD8 + NK1.1+ cells, in mice have been analysed in the lab, and the data showed that these CD8+ NKT subsets might have innate and adaptive immune functions and exhibit stronger cytotoxicity functions than conventional CD8-CD56+ NK and CTL cells; additionally, more granzyme particles were found in their cytoplasm [26]. Second, based on the higher cell proliferation rate in the NKT culture medium that we established, more killer cells can be easily expanded from a small number of seeded PBMCs. Thus, a large number of cells can be used for each transfer treatment. In this protocol, the total cell number of transferred cells in this trial was 1 × 10^10^ cells per week with two transfers, which was significantly greater than the cell numbers in previous studies, which were generally 10^9^ per week or even lower. The safety test data of previous studies in a mouse model (CD8 + NK1.1+ cells), preclinical study and primary clinical safety trial (CD8 + CD56+ cells) in humans showed that this number of CD8 + CD56+ NKT cells caused no obvious adverse reactions or cytotoxicity in recipients (data not shown). Thus, in this study, we continued to use this dose of transferred cells in EGFR-TKI gefitinib combination therapy to further evaluate its safety and efficiency.

The tumour microenvironment in the patient promotes immune suppression and induces anergy and exhaustion in endogenous and transferred immune cells through different mechanisms [35]. Immune checkpoint blockades with anti-PD-1 mAbs successfully reinvigorate TILs and provide clinical benefits to patients with advanced cancer [36]. Unfortunately, published data has shown that anti-PD-1 was not associated with a significantly longer PFS than chemotherapy in NSCLC patients with low PD-L1 expression [37]. Thus, effectively reversing immune suppression in patients with NSCLC is a key factor for successful immunotherapy. Accumulating data on allogeneic transplantation showed that allogeneic human leukocyte antigens (HLAs) induced a T cell response with extraordinary strength and diversity of the alloreactive repertoire compared with classic antigens. These allorecognition-activated alloimmune T cells are the backbone of adaptive immune responses to transplants from donors or to the host, which, respectively cause graft rejection and graft-versus host disease (GVHD) [38, 39]. In this trial, we prepared allogeneic CD8 + CD56+ NKT cells from PBMCs for NSCLC treatment. After transfer, in addition to the direct cellular-mediated cytotoxicity to tumour cells, allogeneic CD8 + CD56+ NKT cells also provide mismatched donor HLA peptide for alloimmune T cell activation in recipients through the allorecognition pathway. Generally, graft-versus-host disease occurs in immune-deficient recipients who undergo immune organ transplantation, such as bone marrow and thymus [40]. However, in this case, there are two points that are dramatically different from haematopoietic stem cells (HSCs) or T cell progenitor transfer treatment. First, transferred allogeneic CD8 + CD56+ NKT cells in this study are mature cells without potential differentiation capability, which stem cells and progenitor cells have. Next, the participants in the trial were immunocompetent, which was different from the patients accepting bone marrow transplantation. Thus, these transferred allogeneic CD8 + CD56+ NKT cells can effectively prime alloimmune T cells of patients to activate cells and reverse tumour microenvironment-induced immune suppression without inducing GVHD. We did not observe any clinical GVHD as an adverse reaction in preclinical and primary clinical studies.

In summary, we developed this clinical trial using allogeneic CD8 + CD56+ NKT cells combined with gefitinib for patients with advanced NSCLC with EGFR mutations to address gefitinib acquired resistance and extend PFS in these patients. Although the sample size is small in this exploratory trial, the success of this approach will help expand the testing scale, extending to other EGFR-TKIs and a variety of other tumours.

## Supplementary Information

**Additional file 1: Table S1.** SPIRIT Checklist.

**Additional file 2: Table S2.** Schedule of Procedures.

## Data Availability

Not applicable – data collection is still ongoing.

## Abbreviations

AE: Adverse event
Anti-PD-1: Anti-programmed death-1
Anti-PD-L1: Anti-programmed death-1 ligand
CAR-T: Chimeric antigen receptor t
DC-CIK: Dendritic cell-cytokine induced killer
CT: Computed tomography
CTCAE: Common Terminology Criteria for AEs
DCR: Disease control rate
EGFR: Epidermal growth factor receptor
EGFR-TKIs: Epidermal growth factor receptor tyrosine kinase inhibitors
GVHD: Graft-versus host disease
HSCs: Haematopoietic stem cells
HLAs: Human leukocyte antigens
LOCF: Last observation carried forward
MDSCs: Myeloid-derived suppressor cells
NKT: Natural killer T
NSCLC: Non-small-cell lung cancer
ORR: Overall response rate
OS: Overall survival
PBMCs: Peripheral blood mononuclear cells
PFS: Progression-free survival
RECIST: Response Evaluation Criteria In Solid Tumors
SPIRIT: Standard Protocol Items: Recommendations for Interventional Trials
TTP: Time to progression
TILs: Tumour-infiltrating lymphocytes
