# Supplementary material for: A phase I/II clinical trial on the efficacy and safety of NKT cells combined with gefitinib for advanced EGFR-mutated non-small-cell lung cancer
Source: BMC Cancer. 2021 Jul 31;21:877. doi: 10.1186/s12885-021-08590-1 (PMC8325186; doi:10.1186/s12885-021-08590-1)
Supplement: Supplementary file 2 — Additional file 2: Table S2. Schedule of Procedures. [file 12885_2021_8590_MOESM2_ESM.doc]

Additional file 2: Table S2: schedule of procedures

| Research stage | | | Screening and Consent | Baseline |  | Allocation | NKT cell treatment period(4 courses of treatment as one cycle, followed by rolling cycle, a total of 3 cycles)^14^ | | | | | | | | | End of treatment | Follow-up period |
| --- | --- | --- | --- | --- | --- | --- | --- | --- | --- | --- | --- | --- | --- | --- | --- | --- | --- |
| Visit^1^ | | | Prior to  registration | After registration and prior to oral gefitinib | 0-8 weeks | week8 | The first four-week course of treatment | | The second four-week course of treatment | | The third four-week course of treatment | | The fourth four-week course of treatment | | Pause for four weeks |  | 12 months, follow-up every 8 weeks |
| Time point | | | - 4 weeks | 0 week | 0-8 weeks | week8 | Day20^15^ | Day23 | Day20 | Day23 | Day20 | Day23 | Day20 | Day23 |  | Day 1 | Day 1 |
| ENROLMENT | Screening data | | X |  |  |  |  |  |  |  |  |  |  |  |  |  |  |
|  | Informed consent | | X |  |  |  |  |  |  |  |  |  |  |  |  |  |  |
|  | Eligibility | | X |  |  |  |  |  |  |  |  |  |  |  |  |  |  |
|  | Randomization | |  |  |  | X |  |  |  |  |  |  |  |  |  |  |  |
| INTERVENTIONS | Arm A | Immune cell collection^2^ |  |  |  | X |  |  |  |  |  |  |  |  |  |  |  |
|  |  | Immune cell reinfusion |  |  |  |  | X | X | X | X | X | X | X | X |  |  |  |
|  |  | Gefitinib(250 mg/day) |  |  | X |  | continuously | | | | | | | | | |  |
|  | Arm B | Gefitinib(250 mg/day) |  |  | X |  | continuously | | | | | | | | | |  |
| ASSESSMENTS | Demographic data^3^ | | X |  |  |  |  |  |  |  |  |  |  |  |  |  |  |
|  | Medical history^4^ | | X |  |  |  |  |  |  |  |  |  |  |  |  |  |  |
|  | Physical examination /Vital signs^5^ | |  | X |  |  | X |  | X |  | X |  | X |  |  | X | X |
|  | KPS | |  | X |  |  | X |  | X |  | X |  | X |  |  | X | X |
|  | Blood routine^6^ | |  | X |  |  | X |  | X |  | X |  | X |  |  | X | X |
|  | Urine routine^7^ | |  | X |  |  | X |  | X |  | X |  | X |  |  | X | X |
|  | Serum biochemistry^8^ | |  | X |  |  | X |  | X |  | X |  | X |  |  | X | X |
|  | Immunological detection^9^ | |  | X |  |  | X |  | X |  | X |  | X |  |  | X | X |
|  | Serum tumor marker detection^10^ | |  | X |  |  | X |  | X |  | X |  | X |  |  | X | X |
|  | Pregnancy test^11^ | |  | X |  |  | X |  | X |  | X |  | X |  |  | X | X |
|  | Electrocardiogram | |  | X |  |  | X |  | X |  | X |  | X |  |  | X | X |
|  | Tumor lesion detection^12^ | |  | X |  |  |  |  | X |  |  |  | X |  |  | X | X |
|  | EORTC QLQ-C30  and EORTC QLQ-LC13^13^ | |  | X |  |  |  |  | X |  |  |  | X |  |  | X | X |
|  | Drug combination | |  | X | X |  | X | X | X | X | X | X | X | X | X | X | X |
|  | Adverse events | |  |  | X |  | X | X | X | X | X | X | X | X | X | X | X |

Note:1.Each visit window period ±3 days. 2.The first collection is arranged on is scheduled the day after randomization. The subsequent collection date is determined according to the actual needs. 3.Demographic data include: age, gender, height, weight, nationality, etc. 4.Medical history data include: past history, present history, history of allergy, concomitant diseases and medication and so on. 5.The vital signs of the sitting position were measured after a quiet rest for 10 minutes. 6. Blood routine: RBC, WBC, HGB, PLT and leukocyte classified count. 7.Urine routine: LEU, ERY, PRO. 8.Serum biochemistry：a.Liver function: ALT, AST, Tbil, GGT, ALP;b.Renal function: Scr. 9.Immunological detection: Immune cell subsets (NKT, NK, CD4+T and CD8+T cells) in peripheral blood were detected. 10.Serum tumor marker detection: CEA. 11.Pregnancy test for women of child bearing potential only. 12.Tumor lesion detection: All clinically recorded tumor sites were examined by CT 11 weeks after the start of gefitinib treatment and every 8 weeks during the treatment period. If tumor remission occurs, repeat the examination after 8 weeks to confirm. 13.After the patient withdrew from the study for reasons other than disease progression, the questionnaire should be completed during the visit period after the end of treatment. 14.During the treatment period, the experimental group(Arm A) and the control group(Arm B) were treated with gefitinib until the disease progressed or completed three cell therapy cycles. 15.Cell reinfusion was carried out on the 20th day and 23rd day, respectively. The patients were infused twice in one course of treatment.
